# The PtdIns 3-Kinase/Akt Pathway Regulates Macrophage-Mediated ADCC against B Cell Lymphoma

**DOI:** 10.1371/journal.pone.0004208

**Published:** 2009-01-16

**Authors:** Trupti Joshi, Latha P. Ganesan, Carolyn Cheney, Michael C. Ostrowski, Natarajan Muthusamy, John C. Byrd, Susheela Tridandapani

**Affiliations:** 1 The Ohio State University Biochemistry Program, The Ohio State University, Columbus, Ohio, United States of America; 2 Division of Pulmonary and Critical Care, Department of Internal Medicine and Comprehensive Cancer Center, The Ohio State University, Columbus, Ohio, United States of America; 3 Division of Hematology-Oncology, College of Medicine, The Ohio State University, Columbus, Ohio, United States of America; 4 Department of Molecular and Cellular Biochemistry, The Ohio State University, Columbus, Ohio, United States of America; Karolinska Institutet, Sweden

## Abstract

Macrophages are important effectors in the clearance of antibody-coated tumor cells. However, the signaling pathways that regulate macrophage-induced ADCC are poorly defined. To understand the regulation of macrophage-mediated ADCC, we used human B cell lymphoma coated with Rituximab as the tumor target and murine macrophages primed with IFNγ as the effectors. Our data demonstrate that the PtdIns 3-kinase/Akt pathway is activated during macrophage-induced ADCC and that the inhibition of PtdIns 3-kinase results in the inhibition of macrophage-mediated cytotoxicity. Interestingly, downstream of PtdIns 3-kinase, expression of constitutively active Akt (Myr-Akt) in macrophages significantly enhanced their ability to mediate ADCC. Further analysis revealed that in this model, macrophage-mediated ADCC is dependent upon the release of nitric oxide (NO). However, the PtdIns 3-kinase/Akt pathway does not appear to regulate NO production. An examination of the role of the PtdIns 3-kinase/Akt pathway in regulating conjugate formation indicated that macrophages treated with an inhibitor of PtdIns 3-kinase fail to polarize the cytoskeleton at the synapse and show a significant reduction in the number of conjugates formed with tumor targets. Further, inhibition of PtdIns 3-kinase also reduced macrophage spreading on Rituximab-coated surfaces. On the other hand, Myr-Akt expressing macrophages displayed a significantly greater ability to form conjugates with tumor cells. Taken together, these findings illustrate that the PtdIns 3-kinase/Akt pathway plays a critical role in macrophage ADCC through its influence on conjugate formation between macrophages and antibody-coated tumor cells.

## Introduction

In recent years, monoclonal antibodies such as rituximab and alemtuzumab [1] have emerged as effective therapeutic agents in the treatment of human malignancies. Although several mechanisms have been proposed for the elimination of tumor cells during antibody therapy, the engagement of Fcγ receptors on immune effector cells plays a dominant role in the clearance of tumor cells during antibody therapy [2]. The population of immune cells that bear Fcγ receptors include monocytes/macrophages, neutrophils and NK cells. However, in a murine model, Uchida et al demonstrated that macrophages are the principal mediators of CD20 antibody-dependent depletion of B cells [3]. There are three classes of FcγR expressed by human and murine macrophages: FcγRI, FcγRIIb and FcγRIII. FcγRI and RIII are activating receptors while FcγRIIb is an inhibitory receptor. In addition, human macrophages express FcγRIIa while murine cells express FcγRIV, both of which are activating receptors [4]–[6].

The mechanism by which macrophages cause the cytolysis of tumor cells depends on the nature of the target tumor cell. Several studies have reported the release of TNFα and nitric oxide (NO) as dominant mediators of macrophage induced cytotoxicity [7]–[11], although the release of certain proteases [12] and reactive oxygen species (ROS) by macrophages has also been reported in few cases [13]. The activation of macrophages for cytolytic function involves a priming event during which macrophages acquire the ability to respond to tumor cells upon subsequent exposure. IFNγ is a potent inducer of macrophage tumoricidal activity [14]–[16]. The priming of macrophages with IFNγ leads to the up-regulation of Fcγ receptors, TNFα, and inducible nitric oxide synthase (iNOS); all of which can enhance the killing of tumor cells [16], [17]. To this end, the administration of *ex-vivo* IFNγ-activated macrophages is being tested as an approach to augment traditional cancer therapies [18], [19].

The signaling pathways involved in NK cell-mediated cytotoxicity have been extensively studied and the activation of protein tyrosine kinases such as lck and Syk kinase are shown to be the initial signaling events in NK cells [20], [21]. The stimulation of both PtdIns 3-kinase and extracellular signal regulatory kinase 1/2 (Erk 1/2) has been shown to play a critical role during NK cell-mediated cytotoxicity [22], [23]. Erk 1/2 control the lytic function of NK cells by regulating the movement of cytosolic perforin and granzyme B towards the target tumor cell [23]. Further it has been shown that Erk 1/2 activation is independent of the traditional Ras-dependent pathway and that PtdIns 3-kinaseinase mediates the activation of Erk 1/2 through sequential involvement of Rac1, PAK1 and MEK [22]. In addition, although the release of cytotoxic granules containing perforin and granzymes by NK cells mediates the lysis of tumor cell, an efficient conjugate formation between the NK cell and the target tumor cell is critical for the process of cytotoxicity [24], [25]. This process involves the formation of immunologic synapse between NK cell and target cell [26], [27]. During the synapse formation, there is a rapid reorganization of the NK cell cytoskeleton including the reorientation of the Golgi complex and the microtubule-organizing center (MTOC) as well as the remodeling of actin cytoskeleton [28]–[30].

As opposed to NK cell cytotoxicity, the signaling events that regulate macrophage mediated ADCC are yet to be characterized. The aim of this study was to identify the signaling pathways that play a crucial role in macrophage response to antibody-coated tumor cells. For this purpose, we have used Rituximab-coated human Burkitt's B cell lymphoma cell line Raji cells as tumor targets. Rituximab is a human-mouse chimeric monoclonal antibody specific for CD20 antigen expressed on B cells. Rituximab has been shown to be effective in the treatment of various forms of B cell malignancies including the aggressive and indolent B cell non-Hodgkin's lymphoma (NHL) as well as B-cell chronic lymphocytic leukemia (CLL) [31]. We demonstrate a critical role for the PtdIns 3-kinase/Akt pathway in macrophage mediated ADCC against tumor cells. We first show that IFNγ primed macrophages mediate Rituximab-dependent killing of B cell lymphoma. The interaction of macrophages with antibody-coated tumor targets leads to the activation of multiple signaling events including the activation of tyrosine kinases and PtdIns 3-kinase/Akt. Pharmacological inhibition of PtdIns 3-kinase/Akt completely abolished macrophage cytotoxicity indicating that the activation of PtdIns 3-kinase/Akt is critical for macrophage cytotoxicity. Consistent with this, murine peritoneal macrophages expressing overactive Akt (Myr-Akt) showed significantly enhanced ADCC compared to their wild-type counterparts. Further analysis of the role of the PtdIns 3-kinase/Akt pathway in macrophage ADCC reveals that this pathway regulates macrophage cytotoxicity at the level of conjugate formation between the effector macrophages and antibody-coated tumor cells.

## Materials and Methods

### Cells, antibodies and reagents

Raw 264.7 cells and Raji cells were obtained from American Type Culture Collection (Manassas, VA) and maintained in RPMI 1640 supplemented with 5% fetal bovine serum. Recombinant mouse IFNγ and TNFα neutralizing antibody were purchased from R&D systems (Minneapolis, MN). All phospho-specific antibodies were from Cell Signaling Technology (Danvers, MA), iNOS antibody was from BD Transduction laboratories (Franklin Lakes, NJ), mouse anti-human CD37 antibody was from BD Biosciences and actin antibody was from Santa Cruz Biotechnology (Santa Cruz, CA). Rituximab and Herceptin antibodies were produced by Genentech (San Francisco, CA). All pharmacological inhibitors were purchased from Calbiochem. Griess Reagent kit was from Molecular Probes (G-7921) (Eugene, OR). Goat anti-mouse Alexa Fluor 594 and FITC-phalloidin were purchased from Molecular probes. TNFα ELISA kit was purchased from R&D systems.

### Isolation of peritoneal macrophages

Transgenic mice with macrophage-specific expression of Myr-Akt have been described earlier [32]. Peritoneal macrophages from transgenic mice and their wild-type littermates were induced by i.p. injection of 1.5 ml of 2.9% Brewer's thioglycolate broth. Macrophages were harvested 4–5 days post-injection by peritoneal lavage using RPMI supplemented with 10% FBS. RBCs were lysed by incubating peritoneal exudate cells with 3–5 ml of RBC lysis buffer (155 mM NH_4_Cl, 10 mM KHCO_3_, 0.1 mM EDTA [pH 8.0]) for 3 minutes on ice.

*ADCC assay:* A ^51^Cr-release assay was performed as described elsewhere [33] with some modifications. Macrophages were plated in 96-well V-bottom tissue culture plates in 10% RPMI medium supplemented with mIFNγ (25 ng/ml) and incubated overnight at 37°C. Raji cells were labeled with ^51^Cr followed by coating with Rituximab. Rituximab was used at a final concentration of 10 µg/ml in all assays except those where dose response with Rituximab was measured. Rituximab-coated, ^51^Cr-labeled Raji targets were added to macrophages at various E∶T ratios. After 8 hours incubation at 37°C, supernatants were harvested and the amount of ^51^Cr released was measured in a gamma counter. The percent relative cytotoxicity was determined as [(experimental cpm−spontaneous cpm)/(total cpm−spontaneous cpm)] *100. For assays involving inhibitors, IFNγ primed macrophages were incubated with the appropriate inhibitors for 30 minutes before addition of target cells. Both, L-NMMA, a competitive inhibitor of nitric oxide production and TNFα neutralizing antibody were added to macrophage cultures along with IFNγ and incubated overnight before addition of targets.

### Preparation of target cells for signaling and cytokine experiments

Raji cells were first coated with Rituximab (10 µg/ml) at 37°C for 20 minutes. Unbound antibody was removed by washing cells once in PBS. Cells were then fixed on ice in 1% paraformaldehyde (PFA) in PBS for 10 minutes. The cells were finally washed with PBS thoroughly to remove PFA. Uncoated or control antibody (Herceptin) treated cells were processed in an identical manner.

### Cell stimulation, lysis and Western blotting

Raw 264.7 cells or peritoneal macrophages were plated in 48-well tissue culture plates and primed with mIFNγ (25 ng/ml) overnight. Cells were then stimulated with fixed Rituximab-coated or uncoated Raji targets at 1∶1 E∶T ratio for indicated time points. Cells were lysed in TN1 lysis buffer (50 mM Tris pH 8.0, 10 mM EDTA, 10 mM Na_4_P_2_O_7_, 10 mM NaF, 1% Triton-X 100, 125 mM NaCl, 3 mM Na_3_VO_4_, 10 µg/ml each aprotinin and leupeptin). Protein-matched whole cell lysates were separated by SDS-PAGE, transferred to nitrocellulose membranes, probed with the antibody of interest and developed with enhanced chemiluminescence (ECL).

### Western Blot Data Quantitation

The ECL signal was quantitated using a scanner and a densitometry program (Scion Image; Scion, Frederick, MD). Background pixel values were subtracted, the signal was normalized to the amount of actin and the normalized band intensity values were plotted.

### Preparation of heat-aggregated IgG (IgG immune-complexes)

Heat-aggregated IgG was prepared according to methods described previously [34]. In brief Chromopure mouse IgG (Jackson ImmunoResearch Laboratories, West Grove, PA) at a concentration of 350 µg/ml was heated at 62°C for 30 minutes, then cooled on ice immediately and used to stimulate cells.

### Measurement of TNFα by ELISA

Raw 264.7 cells or peritoneal macrophages were incubated with or without IFNγ (25 ng/ml) overnight, treated with DMSO or 10 µM Ly294002 and then stimulated with paraformaldehyde-fixed Rituximab-coated Raji targets for 8 hours at E∶T ratio of 1∶1. The supernatants were harvested and analyzed by ELISA. To test the neutralizing ability of TNFα blocking antibody, similar samples were run in parallel in the presence of TNFα blocking antibody.

### Measurement of nitrite by Griess Reagent

The levels of NO were measured by assaying the culture supernatants for NO_2_^−^, a stable product of NO with molecular oxygen. The assay was performed with a modification of previously described method [35] using Griess Reagent kit from Molecular Probes. For the assay, macrophages were plated in RPMI containing no phenol red and no serum. To test the effect of Fc receptor clustering on NO production, macrophages were primed with IFNγ for 16 hours, treated with DMSO or 10 µM Ly294002 for 30 minutes followed by stimulation with immune-complexes (350 µg/ml) for 8 hours (IFNγ was left in the medium during immune-complex stimulation). For nitrite assay, 100 µl of supernatant was mixed with 50 µl of Griess Reagent (Molecular Probes G-7921) and incubated at room temperature in dark for 30 minutes. The absorbance was measured at 520 nm and nitrite concentrations were calculated from a standard curve obtained using standards containing increasing concentration of NaNO_2_.

### Analysis of conjugate formation

IFNγ primed macrophages and Raji tumor cells were stained for analysis of conjugates using a method described elsewhere [27]. The IFNγ primed macrophages were treated with DMSO or inhibitors (10 µM Ly294002 or 20 nM rapamycin) for 30 minutes and then mixed with Rituximab-coated Raji cells at 5∶1 ratio for 30 minutes at 37°C in suspension. The cell suspension was transferred to poly-L-lysine coated cover-slips and the plate was centrifuged at 1600 rpm for 5 minutes. The plate was further incubated at 37°C for 1 hour. The cells were then fixed using 4% formaldehyde, washed twice and incubated with mouse anti-human CD37 antibody overnight followed by Alexa Fluor 594-conjugated goat anti-mouse for 4 hours. The cells were then permeabilized using 0.2% Triton-X100 and stained with FITC-phalloidin.

Cell conjugates were analyzed using Olympus microscope (Olympus, BX40F-3, Melville, NY) and images were captured using digital video camera (Olympus U-CMAD-2, Optronics, Galeta, CA). 100 red cells were analyzed per cover-slip and a total of 300 red cells per condition were scored for conjugate formation. The results were expressed as % conjugates formed.

### Spreading of macrophages on Rituximab-coated surfaces

The spreading of macrophages on antibody-coated surfaces was measured as described elsewhere [36]. Thioglycollate-elicited wild-type peritoneal macrophages were treated with DMSO or 10 µM Ly294002 for 30 minutes at 37°C. They were then allowed to spread on cover-slips pre-coated with 1 mg/ml Rituximab for different time points. The spreading of cells was analyzed using Nikon E800 microscope and images were captured using a Nikon FDX-35 camera. The cells were then fixed with 4% formaldehyde. The surface area occupied by cells was measured using Morphometry software (version 6) and expressed as arbitrary units (AU).

### Statistical Analysis

All data were analyzed using Student's t-test and p value of ≤0.05 was considered significant.

## Results

### IFNγ-primed macrophages show Rituximab-dependent lysis of B cell lymphoma Raji cells

**Figure 1:**
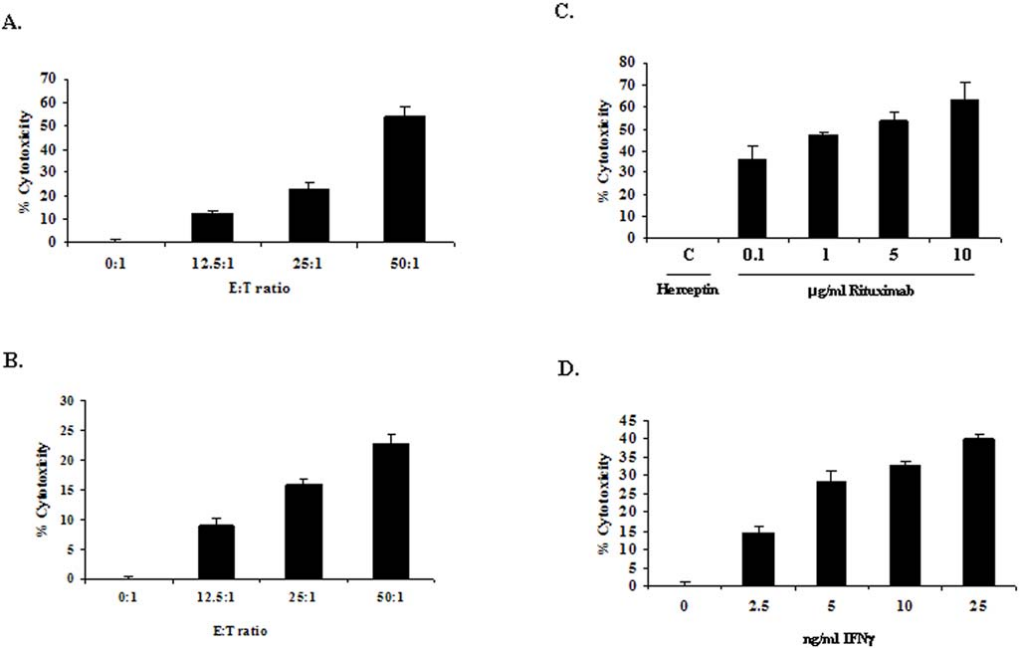
IFNγ-primed macrophages show Rituximab-dependent lysis of B cell lymphoma Raji cells. A. Raw 264.7 cells or B. peritoneal macrophages were primed with 25 ng/ml IFNγ over-night. ^51^Cr- labeled Raji cells coated with 10 µg/ml Rituximab were added to IFNγ primed macrophages at E∶T ratios of 0∶1, 12.5∶1, 25∶1 and 50∶1. The amount of ^51^Cr released was measured at the end of 8 hours and is shown as % cytotoxicity. C. ^51^Cr-labeld Raji cells were coated with increasing concentrations of Rituximab as indicated in the figure or treated with Herceptin (10 µg/ml). They were then co-cultured with IFNγ-primed Raw 264.7 cells for 8 hours. The supernatants were harvested and the amount of ^51^Cr released was measured. The graph shows % cytotoxicity values obtained at 50∶1 E∶T ratio. D. To test the effect of IFNγ priming, Raw 264.7 cells were primed with increasing concentration of IFNγ and co-cultured with ^51^Cr- labeled Rituximab-coated Raji targets for 8 hours. % cytotoxicity values obtained at the E∶T ratio of 50∶1 are shown in the graph.

We first tested whether macrophages could elicit Rituximab-dependent killing of B cell lymphoma cells. For this, we used a standard ^51^Cr-release assay method which has been extensively employed by several authors to measure the cytotoxic potential of NK cells. In these assays, murine macrophages were primed with IFNγ over-night and then co-cultured with ^51^Cr-labeled, Rituximab-coated Raji cells for 8 hours. The amount of ^51^Cr released at the end of 8 hours was measured and is indicated as % cytotoxicity. First, we analyzed whether murine macrophage cell line Raw 264.7 and primary peritoneal macrophages could mediate cytolysis of Rituximab-coated Raji cells. For this, IFNγ-primed macrophages (effectors) were incubated with ^51^Cr-labeled Raji cells (targets) coated with Rituximab (10 µg/ml) at different effector to target (E∶T) ratios. Our results indicate that both Raw 264.7 cells and peritoneal macrophages show increasing cytotoxicity towards Rituximab-coated Raji cells with the increasing E∶T ratio (Figure 1A and 1B).

To confirm that the cytotoxicity seen in the above experiments is indeed antibody-mediated, IFNγ primed Raw 264.7 cells were incubated with ^51^Cr-labeled Raji cells coated with increasing concentration of Rituximab. The results shown in Figure 1C indicate that there was a dose-dependent increase in the cytotoxicity mediated by Raw 264.7. Moreover tumor cells not coated with Rituximab or treated with irrelevant antibody such as Herceptin which does not bind to Raji cells were not lysed by IFNγ primed macrophages. This indicates that the opsonization by antibodies that specifically bind the tumor cells induce their killing by macrophage effectors.

To examine the requirement of priming by IFNγ, macrophages were treated with increasing concentrations of IFNγ. As indicated in Figure 1D, Raw 264.7 cells left untreated with IFNγ could not mediate ADCC while the cytotoxicity of macrophages increased gradually with increasing doses of IFNγ. These data are consistent with the previously reported requirement of IFNγ by macrophages for tumoricidal activity [14], [15].

### Rituximab-coated targets induce significant activation of Syk kinase and the PtdIns 3-kinase/Akt pathway in macrophages

**Figure 2:**
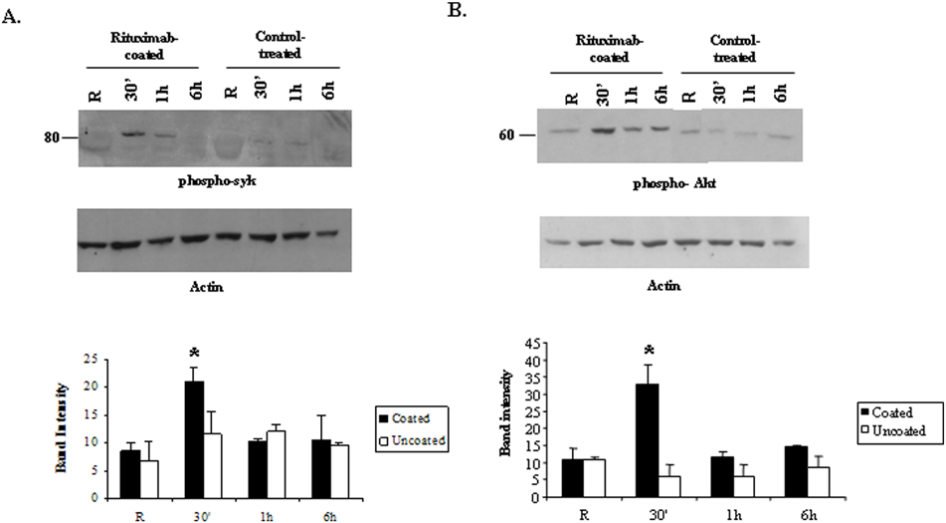
Rituximab-coated targets induce significant activation of Syk kinase and the PtdIns 3-kinase/Akt pathway in macrophages. IFNγ-primed peritoneal macrophages were stimulated with paraformaldehyde-fixed Rituximab-coated or control antibody-treated Raji cells at E∶T ratio of 1∶1 for indicated time points. Protein-matched whole cell lysates were analyzed by Western blotting with A. phospho-Syk antibody (upper) and B. phospho-Akt antibody (upper). The same membranes were reprobed with actin antibody (lower). The graphs in the lower panels show mean and SD of the band intensity of phospho-Syk and phospho-Akt from three independent experiments. Data were analyzed by student's t-test (* = p value≤0.05).

We investigated the activation of several signaling pathways in macrophages in response to antibody-coated tumor targets by stimulating IFNγ-primed peritoneal macrophages with formaldehyde-fixed Rituximab-coated Raji targets for various time points. Similar approaches have been used in the past in the studies of NK cell mediated cytotoxicity [22], [23]. Raji cells incubated with Herceptin (which does not bind to Raji cells) were used as a control. As shown in Figure 2A upper panel, strong phosphorylation of Syk kinase was seen at 30 minutes with Rituximab-coated targets but not with control antibody-treated targets. The graph shown in lower panel is a quantitation of band intensity from three independent experiments. The stimulation of macrophages with Rituximab-coated tumor cells also resulted in the activation of the PtdIns 3-kinase/Akt pathway as seen by serine phosphorylation of Akt while tumor cells incubated with control antibody failed to induce Akt activation (Figure 2B). The graph shown in lower panel represents the average of phospho-Akt band intensity from three experiments. In addition to the activation of these pathways, we also observed an increase in phosphorylation of Erk/MAPK with targets opsonized with Rituximab. No activation of p38 or JNK MAPKs was seen (data not shown). Collectively, these results indicate that when macrophages are incubated with tumor cells coated with the appropriate antibody display the activation of specific signaling pathways.

### Activation of the PtdIns 3-kinase/Akt pathway is critical for macrophage ADCC

**Figure 3:**
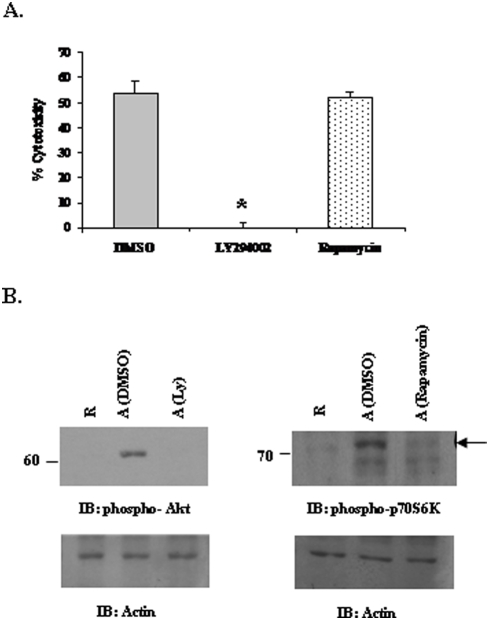
Activation of the PtdIns 3-kinase/Akt pathway is critical for macrophage ADCC. A. IFNγ-primed Raw 264.7 cells were treated with DMSO, 10 µM Ly294002 and 20 nM Rapamycin for 30 minutes. They were then incubated with ^51^Cr-labeled Rituximab-coated Raji cells for 8 hours. The graph shows % cytotoxicity at 50∶1 E∶T ratio. B. To test the specificity of the inhibitors used, Raw 264.7 cells were incubated with DMSO, 10 µM Ly294002 or 20 nM Rapamycin for 30 minutes. The cells were then stimulated with immune-complexes for 7 minutes. Protein-matched cell lysates were analyzed by Western blotting with phospho-Akt antibody and phospho-p70S6K antibody (upper). R indicates resting samples and A indicates cells activated with immune-complexes. The same membranes were reprobed with actin antibody (lower).

The above experiments demonstrated that there is a significant activation of the PtdIns 3-kinase/Akt pathway in macrophages upon ligation with antibody-coated targets. In order to examine whether the activation of the PtdIns 3-kinase/Akt pathway is important during cytotoxicity, we used Ly294002, a specific pharmacological inhibitor of PtdIns 3-kinase. Previous work from our group has demonstrated that Akt promotes Fc receptor-mediated phagocytosis through activation of p70S6 kinase (p70S6K) [32]. To test the requirement of downstream p70S6K in macrophage mediated ADCC, we incorporated rapamycin, an inhibitor of mTOR, in this experiment. IFNγ primed Raw 264.7 cells were pre-treated with either DMSO or 10 µM Ly294002 or 20 nM rapamycin for 30 minutes. They were then co-cultured with ^51^Cr-labeled, Rituximab-coated Raji targets for 8 hours. At the end of 8 hours, supernatants were harvested and the amount of ^51^Cr released was measured. As seen in Figure 3A, pre-treatment of macrophages with PtdIns 3-kinase inhibitor resulted in complete inhibition of antibody-dependent cytotoxicity indicating that the PtdIns 3-kinase/Akt pathway plays a critical role in macrophage response towards antibody-coated targets. On the other hand, the cytotoxic ability of macrophages treated with rapamycin remained unaffected. This suggests that unlike phagocytosis, activation of p70S6K is dispensable during FcγR-mediated macrophage cytotoxicity. To ensure the specificity of the inhibitors, Raw 264.7 cells were first incubated with respective inhibitors for 30 minutes and then stimulated with immune-complex (heat-aggregated IgG) for 7 minutes. The protein-matched whole cell lysates were analyzed using phospho-p70S6K antibody for rapamycin and phospho-Akt antibody for Ly294002. The cells treated with vehicle-control (DMSO) showed significant induction in phosphorylation of Akt (Figure 3B, left) and p70S6K (Figure 3B, right) whereas the treatment of cells with Ly294002 and rapamycin resulted in complete inhibition of phosphorylation of Akt and p70S6K respectively.

### Over-expression of active Akt enhances macrophage ADCC

**Figure 4:**
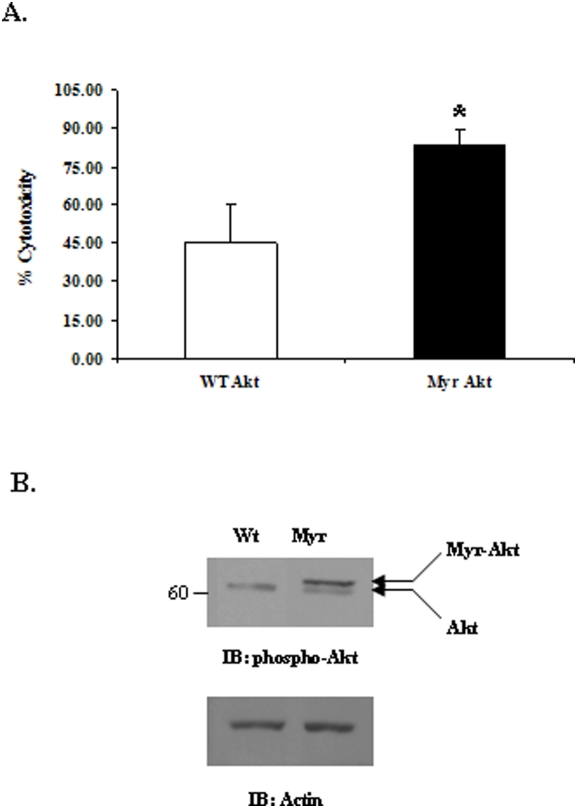
Over-expression of active Akt enhances macrophage ADCC. A. Wild-type and Myr-Akt expressing peritoneal macrophages were activated with IFNγ over-night. ^51^Cr- labeled Rituximab-coated Raji targets were added and supernatants harvested at the end of 8 hours. The graph shows % cytotoxicity values obtained at E∶T ratio of 50∶1. Data was analyzed using student's t-test (* = p value≤0.05). B. To confirm the expression of the Myr-Akt transgene, cell lysates from wild-type and Myr-Akt peritoneal macrophages were analyzed by Western blotting with phospho-Akt antibody (upper). The same membrane was reprobed with actin antibody (lower).

The above data indicated that Rituximab-coated Raji cells stimulate Akt activation in macrophages and activation of PtdIns 3-kinase upstream of Akt is necessary for macrophage ADCC. Also a previous report from our group has demonstrated that constitutively active Akt significantly up-regulates FcγR-mediated phagocytosis by macrophages [32]. To determine whether activation of Akt downstream of PtdIns 3-kinase plays a role during macrophage mediated ADCC, we tested the cytotoxic ability of peritoneal macrophages from previously described transgenic mice over-expressing macrophage specific, constitutively active Akt (Myr-Akt). The results shown in Figure 4A indicate that Myr-Akt macrophages show significantly enhanced cytolysis of Rituximab-coated Raji cells as compared to their wild-type counterparts. As described previously, there is no difference in the expression of FcγR on the surface of Myr-Akt and wild-type macrophages [32] indicating that the enhanced ADCC is indeed a function of over-active Akt and not attributable to the changes in Fc receptor expression. To confirm the expression of Myr-Akt transgene, whole cell lysates from wild-type and Myr-Akt macrophages were resolved on SDS-PAGE and blotted with phospho-Akt antibody (Figure 4B, upper panel). The lower panel of Figure 4B is a reprobe of the same membrane with actin antibody to show equal loading of protein. Together these results demonstrate that downstream of PtdIns 3-kinase, Akt activation plays an important role in macrophage response to antibody-coated tumor cells.

### Influence of the PtdIns 3-kinase/Akt pathway on mediators of cytotoxicity: TNFα and nitric oxide

Several previous studies have indicated that TNFα and nitric oxide (NO) produced by activated monocytes/macrophages are predominant effectors of cytotoxicity [7]–[9]. These studies further suggest that although many cells are sensitive to TNFα-mediated lysis, vast majority of the cells appear to be resistant to TNFα-induced cytolysis [37]–[40]. Having established the role of the PtdIns 3-kinase/Akt pathway in macrophage mediated ADCC, we next wanted to understand the mechanism by which this pathway regulates cytotoxicity. We hypothesized that the PtdIns 3-kinase/Akt pathway may enhance macrophage induced tumoricidal activity by promoting the production of TNFα and/or nitric oxide.

**Figure 5:**
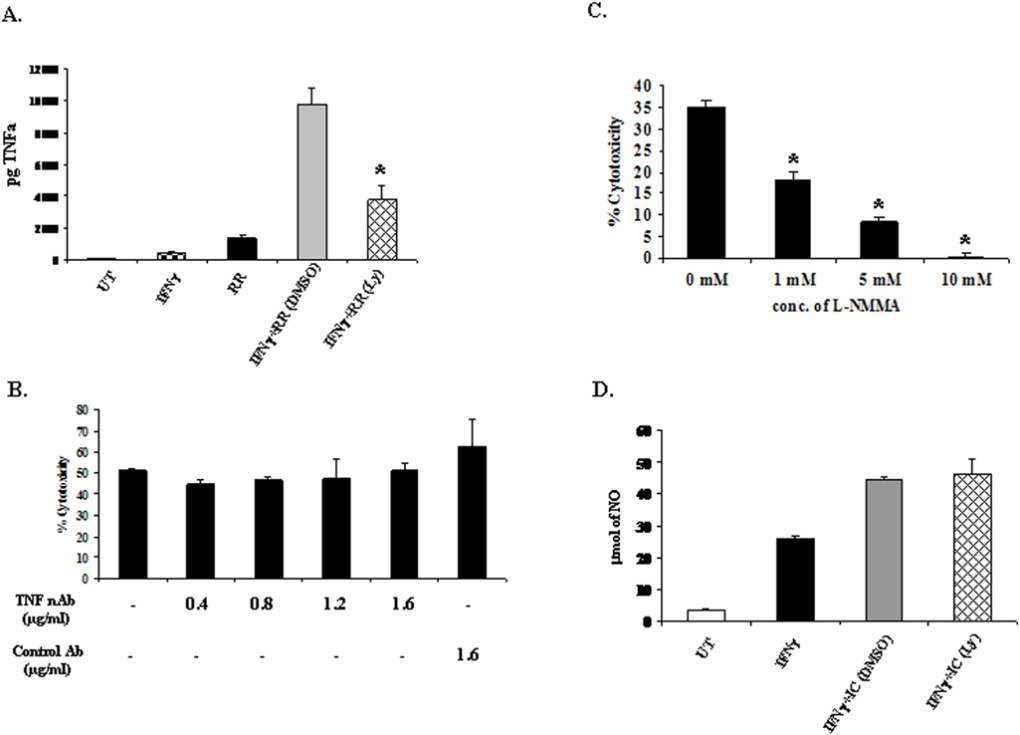
Influence of the PtdIns 3-kinase/Akt pathway on mediators of cytotoxicity: TNFα and nitric oxide. A. Raw 264.7 cells were left untreated or treated with IFNγ for 16 hours. They were then incubated with DMSO or 10 µM Ly294002 for 30 minutes followed by stimulation with Rituximab-coated Raji cells (indicated as RR in the figure) at E∶T ratio of 1∶1 for 8 hours. Controls conditions consisted of Raw 264.7 cells stimulated with media alone (UT) or IFNγ (25 ng/ml) alone or Rituximab-coated Raji cells alone. Supernatants were harvested and levels of TNFα were measured using ELISA. B. Raw 264.7 cells were cultured in presence of IFNγ and increasing concentrations of TNFα neutralizing antibody (0–1.6 µg/ml) or control antibody (1.6 µg/ml). After 16 hours of IFNγ priming, ^51^Cr- labeled Rituximab-coated Raji targets were added to Raw 264.7 cells and incubated for 8 hours. The graph shows % cytotoxicity at E∶T ratio of 50∶1. C. Raw 264.7 cells were primed with IFNγ in presence of varying concentrations of L-NMMA (0–10 mM). Next day ^51^Cr- labeled Rituximab-coated Raji targets were added and amount of ^51^Cr released was measured at the end of 8 hours. The graph shows % cytotoxicity at E∶T ratio of 50∶1. D. Raw 264.7 cells were pre-treated with IFNγ (25 ng/ml) for 16 hours. They were then stimulated with immune-complex (indicated as IC in the figure) at concentration of 350 µg/ml for 8 hours in presence of DMSO or 10 µM Ly294002. Controls conditions consisted of Raw 264.7 cells cultured with media alone (UT) or IFNγ (25 ng/ml) alone. Supernatant were harvested at the end of 24 hours and levels of NO produced were measured.

To test this hypothesis, we measured the amount of TNFα produced by IFNγ-primed macrophages in response to antibody-coated tumor cells in presence or absence of PtdIns 3-kinase/Akt inhibitor. For these experiments, Raw 264.7 cells were primed with IFNγ (25 ng/ml) for 16 hours, treated with either DMSO or 10 µM Ly294002 for 30 minutes and then stimulated with formaldehyde-fixed Rituximab-coated Raji cells at E∶T ratio of 1∶1 for 8 hours. Raw 264.7 cells left untreated with IFNγ, or stimulated with IFNγ alone or Rituximab-coated Raji cells alone served as control conditions for the experiment. Supernatants were harvested and levels of TNFα were measured using ELISA. Consistent with previous reports, the results shown in Figure 5A indicate that macrophages primed with IFNγ alone produced a small amount of TNFα [17], [41]. Further activation of IFNγ-primed macrophages with antibody-coated tumor cells (indicated as RR in the figure) resulted in significantly higher levels of TNFα in presence of DMSO. However, TNFα production stimulated by ligation with antibody-coated tumor cells was significantly inhibited in IFNγ primed macrophages treated with Ly294002 indicating that the PtdIns 3-kinase/Akt pathway is involved in FcγR-mediated TNFα production.

As mentioned earlier, majority of tumor cells have been shown to be resistant to TNFα-mediated lysis [37]–[40]. Therefore, we next tested the requirement of TNFα in cytolysis of Rituximab-opsonized Raji cells by incorporating TNFα neutralizing antibody in the cytotoxicity assays. As seen in Figure 5B, incubation of macrophages with TNFα neutralizing antibody had no effect on their cytotoxic ability towards Rituximab-coated Raji cells suggesting that Raji cells are resistant to lysis by TNFα. We also confirmed the blocking activity of TNFα neutralizing antibody in a parallel experiment using ELISA (data not shown). Thus, although PtdIns 3-kinase/Akt regulates TNFα production, TNFα is not required for cytolysis of Raji cells.

Next, we determined the role of NO in macrophage ADCC against Raji cells. Resting macrophages do not produce nitric oxide since inducible nitric oxide synthase (iNOS), the enzyme involved in NO synthesis by macrophages, is not constitutively expressed [42], [43]. However, priming of macrophages with IFNγ induces the expression of iNOS and thereby leads to NO production [16]. Since L-arginine is used as a substrate by iNOS for NO synthesis, analogs of L-arginine such as L-NMMA act as competitive inhibitors of iNOS activity and thereby block NO production [44]. To examine the role of NO in lysis of Raji cells, we measured the percent cytotoxicity of macrophages in the presence of increasing concentrations of L-NMMA. The results shown in Figure 5C demonstrate that macrophages treated with L-NMMA lose their cytolytic ability towards Rituximab-coated Raji cells in a dose-dependent manner. These data demonstrate that nitric oxide is necessary for lysis of Rituximab-coated Raji cells by macrophages.

Having established the requirement for NO production, we next analyzed the role of PtdIns 3-kinase in NO production. Here, Raw 264.7 cells were primed with IFNγ (25 ng/ml) alone or further stimulated by IgG immune-complexes in presence of either DMSO or 10 µM Ly294002 for 8 hours and the amount of NO present in supernatants was measured. As shown in Figure 5D, macrophages when primed with IFNγ produced NO. NO production was further enhanced significantly following the activation of Fcγ receptors by immune-complexes. However, inhibition of PtdIns 3-kinase/Akt had no effect on the production of NO upon FcγR clustering indicating that PtdIns 3-kinase/Akt plays no role in FcγR-induced NO production by macrophages.

These results can be summarized as follows: a) FcγR-mediated TNFα production is regulated by PtdIns 3-kinase/Akt; however, TNFα is not involved in lysis of Rituximab-coated Raji cells, b) Although nitric oxide is required for macrophage ADCC against Raji cells, activation of PtdIns 3-kinase/Akt does not contribute to NO synthesis.

### The PtdIns 3-kinase/Akt pathway promotes conjugate formation between macrophages and tumor cells

**Figure 6:**
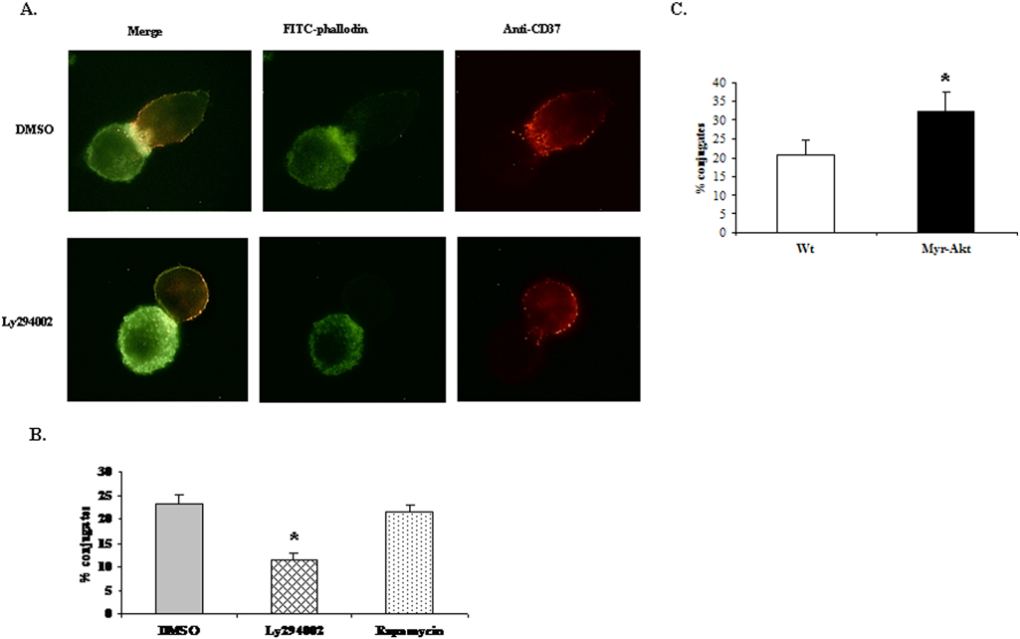
The PtdIns 3-kinase/Akt pathway promotes conjugate formation between macrophages and tumor cells. A. Raw 264.7 cells were primed with IFNγ overnight followed by treatment with DMSO or 10 µM Ly294002 or 20 nM Rapamycin for 30 minutes. They were then mixed with Rituximab-coated Raji cells at the E∶T ratio of 5∶1 and adhered to poly-L-lysine coated cover-slips for 1 hour. Raji cells were labeled with CD37 antibody followed by anti-mouse Alexa Fluor 594 (red fluorescence). F-actin was labeled with FITC-phalloidin. Stained cells were observed under the microscope for action polarization at the synapse. B. Samples were processed as described in A. 100 red cells were analyzed per cover-slip and a total of 300 red cells per condition were scored for conjugate formation. The results were expressed as % conjugates formed. The data indicate % conjugates formed in one experiment. Similar results were obtained in three independent experiments. C. Conjugate formation by WT and Myr-Akt expressing peritoneal macrophages was determined by processing the samples as described in A and B.

The above results suggest that an event other than the release of cytotoxic mediators is a requisite for the cytotoxicity and is probably influenced by PtdIns 3-kinase/Akt pathway. Many studies have demonstrated that in the case of NK cell cytotoxicity, the process of conjugate formation between the effector NK cell and the target tumor cell is critical and determines the efficacy of killing [24], [25]. The two important events involved in conjugate formation include reorganization of the actin cytoskeleton and polarization of microtubule organizing center (MTOC) at the synapse [28], [29], [45]. It has been shown that actin remodeling is required for the maintenance of conjugate formation and also for the localized release of cytotoxic mediators [46], [47]. Several other reports have demonstrated the role of PtdIns 3-kinase in cytoskeletal rearrangements [48]–[50]. We therefore analyzed whether the loss in macrophage ADCC upon inhibition of PtdIns 3-kinase/Akt was due to defects in conjugate formation with tumor cells. To visualize the conjugates, we allowed macrophages (pre-treated with DMSO, Ly294002 or rapamycin) and tumor cells mixed at E∶T ratio of 5∶1 to adhere to poly-L-lysine coated cover-slips. Rituximab-coated Raji tumor cells were then labeled with mouse anti-human CD37 antibody (a B cell-specific cell surface marker) followed by goat anti-mouse Alexa Fluor 594 (red fluorescence) and IFNγ primed macrophages were stained with FITC-phalloidin (green fluorescence). The visualization of actin polarization at the junction between the macrophage and the Rituximab-coated Raji cell indicated that DMSO treated macrophages showed polarization of actin at the synapse formed with tumor cell, whereas Ly294002 treated macrophages failed to show significant actin polarization (Figure 6A). To measure the number of conjugates, 300 tumor cells were counted for each condition and were scored for conjugate formation. The experiment was repeated at least three times. As shown in Figure 6B, pre-treatment of macrophages with Ly294002 reduced their ability to form conjugates with tumor cells. There was no reduction in percent conjugates formed upon rapamycin treatment. Similar experiments were performed using peritoneal macrophages isolated from WT and Myr-Akt mice. Myr-Akt macrophages displayed significantly increased conjugate formation with antibody-coated tumor cells (Figure 6C). These data indicate that the PtdIns 3-kinase/Akt pathway plays a role in promoting the contact between macrophages and antibody-coated tumor cells.

### The PtdIns 3-Kinase/Akt pathway enhances the spreading of macrophages on antibody-coated surfaces

**Figure 7:**
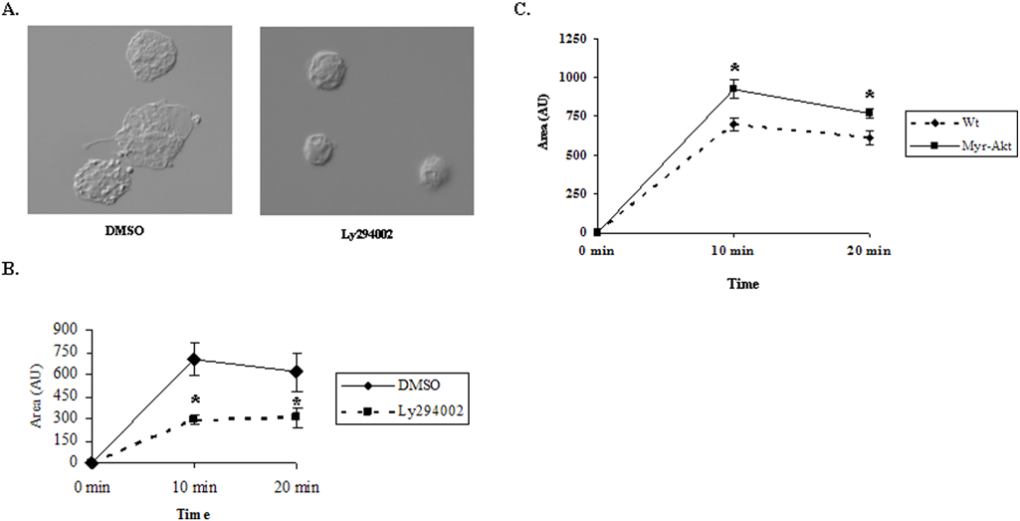
The PtdIns 3-Kinase/Akt pathway enhances the spreading of macrophages on antibody-coated surfaces. A. Spreading of thioglycollate-elicited peritoneal macrophages on Rituximab-coated cover-slips in the presence of DMSO or 10 µM Ly294002. B. Peritoneal macrophages, pre-incubated with DMSO or 10 µM Ly294002 were allowed to spread on Rituximab-coated cover-slips at 37°C for indicated times before fixation. Data are presented as mean surface area occupied by cells. Shown are data from one experiment. Similar results were obtained from three independent experiments. C. Mean surface area occupied by Wt and Myr-Akt peritoneal macrophages.

As an alternative approach, we analyzed the ability of macrophages to spread on Rituximab-coated surfaces. Earlier studies have indicated that spreading of macrophages on human IgG-coated surfaces involves the insertion of membrane from intra-cellular source and is dependent upon PtdIns 3-kinase [36]. When we compared the surface area occupied by peritoneal macrophages treated with DMSO or Ly294002 on Rituximab-coated surfaces, it was clearly evident that the spreading of macrophages upon inhibition of PtdIns 3-kinase was restricted to a significant extent (Figure 7A). The surface area occupied by these cells was measured using Morphometry software and showed a significant difference in the areas occupied by DMSO treated cells as opposed to those treated by Ly294002 (Figure 7B). On the other hand, Myr-Akt macrophages occupied a significantly greater surface area as compared to WT macrophages on Rituximab-coated surfaces (Figure 7C).

Taken together, these findings illustrate that the PtdIns 3-kinase/Akt pathway plays a critical role in macrophage response to Rituximab-coated tumor cells at least in part through its influence on conjugate formation between the macrophages and the target tumor cells.

## Discussion

In this report, we demonstrate that the activation of Akt by PtdIns 3-kinase plays an important role in macrophage mediated ADCC against Rituximab-coated Raji cells. The PtdIns 3-kinase/Akt pathway was found to be required for efficient conjugate formation as well as spreading of macrophages on antibody-coated surfaces. In the case of NK cells, studies have shown that conjugate formation is a critical step for the lysis of tumor cells [24], [25]. Our findings similarly indicate that cytoskeletal rearrangements and thereby conjugate formation constitute a pre-requisite for effective cytolysis of antibody-coated tumor cells by monocytes/macrophages as well.

PtdIns 3-kinase regulates a diverse array of cellular activities such as cell growth, survival, cytoskeletal changes and trafficking of intra-cellular organelles. The activation of PtdIns 3-kinase leads to changes in the actin cytoskeleton through the activation of various downstream proteins. PtdIns 3-kinase-dependent activation of Vav leads to the activation of Rac1 which has been shown to be involved in actin reorganization [51], [52]. During FcγR-mediated phagocytosis, our group has demonstrated that activation of Akt downstream of PtdIns 3-kinase up-regulates phagocytic process through the activation of p70S6 kinase [32]. Arf6 is reported to be essential for actin assembly during FcγR-mediated phagocytosis as well as for NK cell ADCC and the activation of Arf6 depends on PtdIns 3-kinase [26], [53]. Further it has been shown that PtdIns 3-kinase activity is also involved in the regulation of membrane availability during engulfment of large size particles [36], [54]. In this report, we show that PtdIns 3-kinase activation during macrophage response to antibody-coated tumor cells leads to the activation of Akt and thereby regulates the cytotoxic response at least in part by promoting effective contact between macrophages and tumor cells. In addition to the activation of Akt, PtdIns 3-kinase may also be involved in the regulation of monocyte/macrophage cytotoxicity through its influence on other proteins involved in actin remodeling as discussed earlier. Further studies are required to test the possible involvement of PtdIns 3-kinase with respect to these pathways.

The role of PtdIns 3-kinase during actin remodeling is well established. However, few studies report the involvement of Akt as a modulator of cytoskeletal changes. Studies in chicken embryonic fibroblasts indicated that activation of Akt by PtdIns 3-kinase induced actin reorganization promoting cell migration [55]. In the same study, authors demonstrated that constitutive activation of Akt was sufficient to activate actin assembly and resulted in increased cell migration even in the presence of Ly294002. A previous study from our group showed that constitutively active Akt increased the phagocytic efficiency of macrophages suggesting that over-active Akt enhances the cytoskeletal rearrangement [32]. In both studies, it was further demonstrated that Akt stimulates actin cytoskeleton through p70S6 kinase. Interestingly, our findings reported here show that inhibition of p70S6K by rapamycin has no effect on the ability of macrophages to lyse Rituximab-coated B cells. This suggests that Akt can modulate actin assembly in other ways. Recently, it has been shown that Akt directly interacts with actin leading to phoshorylation of actin at Akt consensus sites in Ly294002-dependent manner [56]. Akt has been shown to associate with and phosphorylate yet another protein involved in actin remodeling called Girdin [57].

IFNγ is known to be a potent activator of macrophage tumoricidal activities [14], [15]. The treatment of macrophages with IFNγ has been reported to stimulate TNFα and nitric oxide production by macrophages [16], [17]. Our data indicate that activation of FcγR upon IFNγ priming results in enhanced TNFα and nitric oxide production. Fc receptor clustering is known to induce TNFα production even in absence of IFNγ priming. Thus synergistic increase in TNFα production upon co-stimulation with IFNγ and antibody-coated tumor cells was not surprising. However, to our knowledge, this is the first report demonstrating enhanced NO production by IFNγ-primed macrophages when stimulated further by immune-complexes.

The inability of IFNγ-primed macrophages to lyse B cells in the absence of antibody opsonization (Figure 1C) has several implications. It indicates that FcγR clustering on macrophages by Rituximab-coated B cells is an important event and contributes to the conjugate formation with tumor cells. The priming of macrophages by IFNγ alone leads to the production of nitric oxide, which is essential for the cytotoxicity towards B cells. However, mere release of nitric oxide is not sufficient for cytolysis of B cells if they are not occupied by a specific antibody. The activation of the PtdIns 3-kinase/Akt pathway by antibody-coated tumor cells results in stable ligation of tumor cells with macrophages thereby allowing the killing of targets by nitric oxide.

Collectively our findings demonstrate a critical role for the PtdIns 3-kinase/Akt pathway in macrophage-mediated cytotoxicity against Rituximab-coated B cell lymphomas. We show that the PtdIns 3-kinase/Akt pathway is required for occupation of greater surface areas on antibody-coated surfaces. Moreover, the formation of efficient cytolytic synapse between macrophages and Rituximab-coated B cells is completely dependent upon the PtdIns 3-kinase/Akt pathway.
